# High-Phosphate-Induced Hypertension: The Pathogenic Role of Fibroblast Growth Factor 23 (FGF23) Signaling in Sympathetic Nervous System Activation

**DOI:** 10.3390/ijms27031138

**Published:** 2026-01-23

**Authors:** Han-Kyul Kim, Orson W. Moe, Wanpen Vongpatanasin

**Affiliations:** 1 Department of Internal Medicine-Cardiology Division-Hypertension Section, University of Texas Southwestern Medical Center, Dallas, TX 75390, USA; 2 Charles and Jane Pak Center for Mineral Metabolism and Clinical Research, University of Texas Southwestern Medical Center, Dallas, TX 75390, USA; 3 Department of Internal Medicine-Nephrology Division, University of Texas Southwestern Medical Center, Dallas, TX 75390, USA; 4 Department of Physiology, University of Texas Southwestern Medical Center, Dallas, TX 75390, USA

**Keywords:** dietary phosphate, exercise pressor reflex, fibroblast growth factor 23, fibroblast growth factor receptor, sympathetic nervous system, hypertension

## Abstract

Hypertension remains a major public health concern globally. Accumulating evidence suggests that dietary phosphate (Pi) and fibroblast growth factor 23 (FGF23), a phosphaturic hormone, are involved in blood pressure regulation. Experimental studies have shown that excess Pi consumption, largely from inorganic Pi used as a preservative or flavor enhancer in processed foods, and increased FGF23 may contribute to vascular abnormalities, thereby promoting hypertension. Importantly, recent animal studies have demonstrated that peripheral FGF23 can cross the blood–brain barrier and stimulate FGF receptor 4 (FGFR4)-calcineurin signaling in the brain, contributing to sympathetic overactivation and hypertensive responses during high Pi loading. Additionally, dietary Pi loading leads to suppression of Klotho, which may further contribute to hypertension. Such mechanisms are potentially relevant to chronic kidney disease (CKD), a condition characterized by Pi retention, massively elevated FGF23, sympathetic overactivity, and hypertension. This review highlights current evidence linking Pi-induced FGF23 pathogenically to hypertension, with focus on FGF23 translocation to and FGFR4 signaling in the central nervous system as a potential mechanism and therapeutic target for hypertension associated with high Pi intake and CKD.

## 1. Introduction

Hypertension is a major public health concern worldwide. According to the 2023 World Health Organization global report on hypertension, approximately 1.3 billion people are affected globally [1]. Despite clinical advances, only 18–23% of individuals with hypertension achieve blood pressure (BP) of less than 140/90 mmHg with antihypertensive medications. Since the prevalence of hypertension is projected to increase due to growth and aging of the population, effective strategies in the prevention and treatment of hypertension are urgently needed to reduce its burden and associated complications.

Sodium is the key dietary electrolyte implicated in the pathogenesis of hypertension [2]. Reducing dietary salt intake, coupled with potassium supplementation, has been recommended not only to lower BP but also to help prevent cardiovascular events [1,3]. However, a growing body of evidence suggests that other dietary components, such as inorganic phosphate (Pi) [4], may independently promote development of hypertension.

Dietary Pi is widely available from plant- and animal-derived products, as well as additives during food processing [5]. The natural sources of dietary Pi include grain, fish, dairy, and meat [6]—in fact, anything that contains cells. Plant-derived organic Pi, which is dominated by non-absorbable phytates, has the lowest oral bioavailability, followed by animal-derived organic Pi [6]. Pi additives, used increasingly as preservatives, flavor enhancers, emulsifiers, stabilizers, leavening agents, texture enhancers, and moisture retainers, are inorganic in composition, which do not require intestinal hydrolysis and release, and thus have very high oral bioavailability and have become a common universal hidden ingredient in all processed food [6,7,8,9]. Furthermore, regulatory agencies do not require food manufacturers to disclose added Pi. As a result, dietary Pi intake in many countries worldwide is far above the recommended daily allowance [10,11,12]. For example, in the U.S., the recommended daily allowance of Pi intake is 700 mg, whereas the estimate average of daily Pi intake is approximately 1400 mg [13]. It raises concerns about its potential impact on many aspects of health, including the development of hypertension.

In this review, we provide an overview of how Pi loading induces elevated BP with a focus on a specific phosphaturic hormone and its downstream pathways. In particular, we highlight recent experimental studies providing the first direct evidence that the bone-derived phosphaturic hormone fibroblast growth factor 23 (FGF23) can act on the central nervous system (CNS), thereby linking dietary Pi, FGF23 signaling, sympathetic overactivation, and hypertension. This model introduces a novel pathophysiologic paradigm that has not been previously described and serves the primary objective of this review.

## 2. Pi and Blood Pressure

Previous prospective studies support the potential BP-raising effects of Pi in normal healthy young adults. In a crossover-controlled study of healthy young male volunteers, a high-Pi diet for 5 days increased systolic BP compared to low- or normal-Pi diet [14]. In a randomized study of healthy young males and females, high Pi consumption for 6 weeks increased 24 h ambulatory BP (ABP), compared to low Pi consumption [15].

Population-based studies, however, showed less consistent results. A cross-sectional study conducted in Black adults found no evidence for an association between dietary Pi intake based solely on a food frequency questionnaire and 24 h ABP [16]. A systematic review of randomized trials and observational studies found positive, negative, or no correlation between dietary Pi intake and BP [17]. Again, dietary recall, rather than 24 h urinary Pi excretion, was used to quantify Pi intake in epidemiological studies. In addition, randomized clinical trials altered Pi intake by increasing or decreasing Pi intake, which introduced changes in other mineral or nutritional consumption beyond Pi alone. When the amount of Pi additive was calculated based on the percent of phosphate (PO_4_) and diphosphorus pentoxide (P_2_O_5_) in package ingredients, a significant association between added Pi and systolic and diastolic BP was observed [18]. These findings highlight the potential role of Pi, commonly found in food additives, on BP and cardiovascular health. Additional studies have shown association between high dietary Pi with multiple cardiovascular outcomes, which may be partially related to hypertension in subjects with and without chronic kidney disease (CKD). In the Cholesterol And Recurrent Event (CARE) study of post-myocardial infarction patients, all-cause death, coronary death, and new congestive heart failure were all positively associated with serum Pi [19,20]. In the Coronary Artery Risk Development in Young Adults (CARDIA), serum Pi was associated with coronary disease in young adults [20,21]. Moving on to intermediate phenotypes, in the Multi-Ethnic Study of Atherosclerosis (MESA), dietary Pi intake was associated with arterial stiffness and left ventricular mass [20,22,23]. While associations never prove causality, much more convincing data are found in animal experiments.

Multiple animal studies support the deleterious role of a high-Pi diet on BP [24,25,26]. Not only was the resting BP affected by dietary Pi loading, we also found exaggerations in arterial BP during hindlimb muscle contraction (i.e., simulated exercise) in rats [25]. We showed that the abnormal pressor responses to exercise are mediated, in part, by an overactive reflex arising in the contracting skeletal muscle, known as the exercise pressor reflex (EPR) [25]. Normally, the EPR is evoked by two mechanisms. Activation of thin fiber muscle afferents traditionally classified as metaboreceptors, which are activated slowly during ischemic muscle contraction (i.e., metaboreflex), and mechanoreceptors, which respond quickly to muscle deformation (i.e., mechanoreflex). The hypertensive response to EPR overactivity during excess Pi consumption was mediated by enhanced activation of both the mechanoreflex, induced by passive hindlimb muscle stretch and the metaboreflex, evoked by intraarterial (IA) capsaicin administration in the hindlimb.

Overall, high dietary Pi intake contributes to BP elevation and hypertensive responses, which are characterized by central sympathetic excitation during physical stress. More recently, high Pi intake has been linked to anxiety phenotype in mice, which may further exacerbate BP during normal activity [27]. Dietary Pi loading triggers physiologic release of the primary phosphaturic hormone FGF23 from osteocytes and osteoblasts to minimize Pi overload [28]. We considered the possibility that FGF23 may play a role in BP regulation during dietary Pi excess. This will be further discussed.

## 3. Mechanisms Underlying High Pi-Induced Hypertension

### 3.1. Role of FGF23 in Sympathetic Nervous System

Accumulating evidence supports a potential role for FGF23 in the development of hypertension. In population-based studies, excess circulating FGF23 levels have been associated with elevated BP or incident hypertension independent of renal function [29,30,31]. Dietary Pi loading elevates circulating FGF23 levels to promote urinary Pi excretion in humans and animals [32,33]. Based on rodent data, dietary Pi loading is hypothesized to increase renal production of glycerol-3-phosphate (G-3-P), which circulates to the bone to trigger FGF23 production [34].

**Figure 1:**
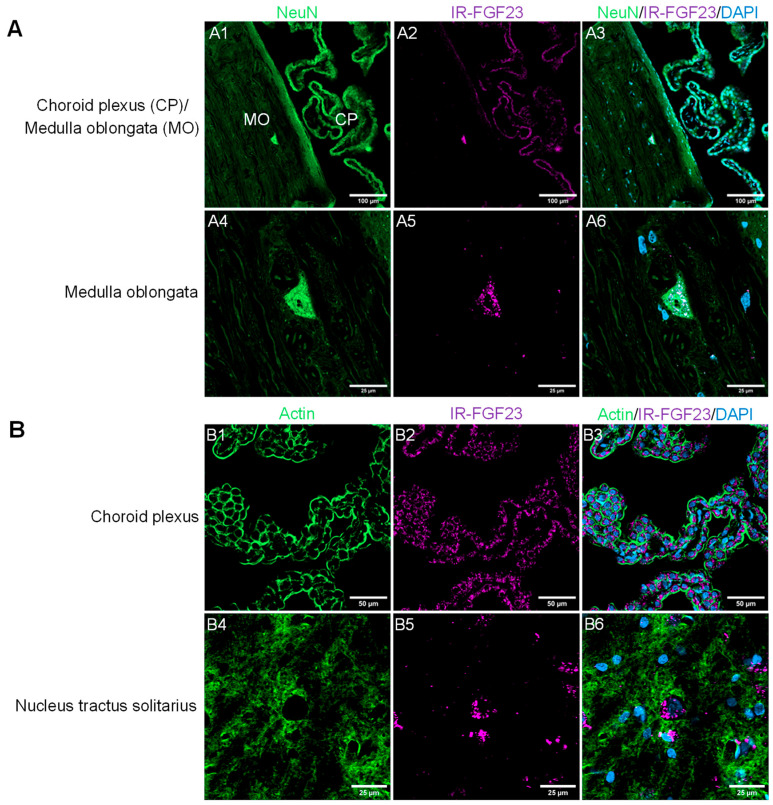
Immunofluorescence images (**A**,**B**) showing the appearance of intravenously injected, infrared-labeled recombinant fibroblast growth factor 23 (IR-FGF23) in choroid plexus (**A2**,**A3**,**B2**,**B3**), medulla oblongata (**A2**,**A3**,**A5**,**A6**), and the nucleus tractus solitarius (**B5**,**B6**) in normal rats ((**A1**–**A6**), midsagittal section view; (**B1**–**B6**), coronal section view). DAPI, 4’,6-diamidino-2-phenylindole. NeuN, neuronal nuclei. Prepared for this review, and different from those in [33].

Mechanistic support for a central role of FGF23 is provided by evidence of its accessibility to the CNS. FGF23 protein has been detected in human cerebrospinal fluid (CSF) [35,36,37,38] and in rat CSF, choroid plexus, and brain regions [39], although the site and even evidence of FGF23 mRNA expression in the CNS has been inconsistent [39,40,41]. Notably, RNA sequencing databases from the Genotype-Tissue Expression (GTEx) RNA-seq dataset do not support FGF23 mRNA expression in the human brain. This limits the interpretation that FGF23 is locally produced within the CNS. In a rat model of dietary Pi loading, FGF23 protein levels increased in the serum, CSF, cerebral cortex, and brainstem without increased brain FGF23 mRNA expression [33]. Furthermore, intravenous administration of infrared-labeled recombinant FGF23 into normal rats has resulted in its rapid appearance in CSF, choroid plexus, and brainstem autonomic control areas (Figure 1), such as the nucleus tractus solitarius (NTS) and the rostral ventrolateral medulla (RVLM) [33]. Together, these findings suggest that circulating FGF23 can enter the CNS by crossing the blood–brain barrier.

In experimental settings, FGF23 has been shown to exert functional effects in the brain. In vitro, FGF23 stimulates neuronal activity in the rat RVLM [42], and consistent with this, intracerebroventricular (ICV) injection of FGF23 in normal rats increased arterial BP and renal sympathetic nerve activity (SNA) in a dose-dependent manner [33]. Collectively, these results support the model of peripheral FGF23 translocation into the brain during dietary Pi loading, contributing to sympathetic activation.

Data on transport routes of FGF23 from the circulation to the brain remain unknown. Interestingly, in vitro studies using human brain microvascular endothelial cells have shown that a high Pi milieu downregulates tight junction proteins, including zona occludens-1, occludin, and claudin-5, which play major roles in the blood–brain barrier integrity [43]. This raises the possibility that a high-Pi environment disrupts the blood–brain barrier structure, potentially facilitating the transfer of circulating FGF23 to the CNS. However, direct evidence for Pi modification of FGF23 trafficking to sympathetic outflow changes remains to be established.

### 3.2. FGF Receptors Mediating the FGF23 Effect

**Figure 2:**
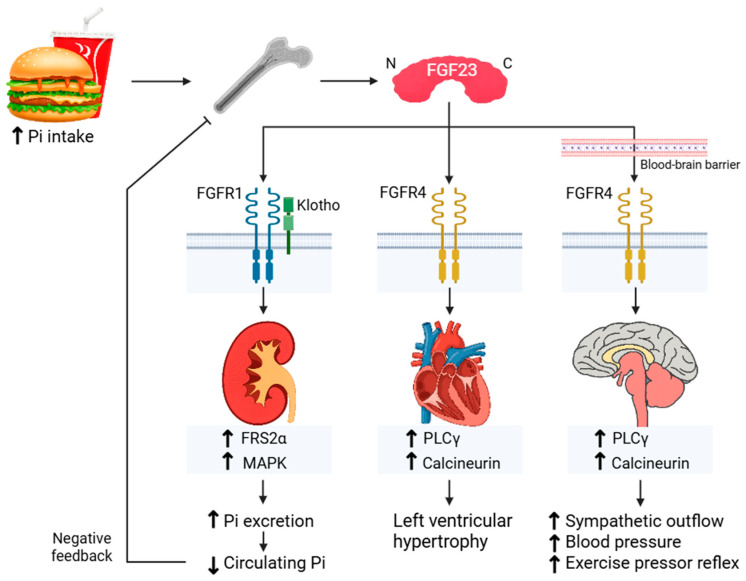
A schematic illustration of the proposed model of fibroblast growth factor 23 (FGF23)-FGF receptors (FGFRs) signaling in high-phosphate (Pi)-intake-induced enhancement of phosphaturia, cardiac remodeling, and hypertension. C, C-terminal region; N, N-terminal region. An upward bold arrow indicates an increase, whereas a downward bold arrow indicates a decrease.

Effects of FGF23 are mediated by binding to the family of FGF receptors (FGFRs), primarily FGFR subtypes 1, 3, and 4 and each of their cognate splice variants (1b, 1c, 2b, 2c, 3b, 3c, 4) [44,45,46,47]. In the kidneys, FGF23 activates mainly FGFR1 in conjunction with coreceptor α-Klotho (and to a lesser extent FGFR4) and ERK1/2 signaling, which inhibits Pi reabsorption, resulting in phosphaturia [45,46]—the canonical pathway (Figure 2). FGFR3 does not modulate the renal effects of FGF23 [44,45,47]. In other organs, such as the heart, FGF23 has been proposed to act via FGFR4, possibly contributing to left ventricular hypertrophy (LVH) via activation of a PLCγ-calcineurin-dependent pathway [48,49,50,51] that theoretically may not require α-Klotho as a co-receptor (noncanonical pathway) (Figure 2). Thus far, structural analysis of the FGF23/FGFR4/α-Klotho/Heparan sulfate complex does not support α-Klotho-independent binding of FGF23 to FGFR4 [52]. This discrepancy may partly reflect differences in organ-specific roles for the coreceptor α-Klotho; thus, such a noncanonical pathway model warrants further investigation.

Building on evidence that FGFRs mediate the peripheral effects of FGF23, recent animal studies from our group have identified FGFR signaling within the CNS that contributes to the central effects of FGF23 on sympathetic and BP responses after excess Pi intake [33]. ICV blockade of a pan-FGFR(1–4) signaling attenuated high-Pi-diet-induced sympathetic overactivation and hypertensive responses during muscle contraction. In contrast, interventions targeting canonical pathways, such as inhibition of FGFR1–3 or disruption of FGF23/FGFR/α-Klotho complex formation using the C-terminal FGF23 peptide had no effect, ruling out contributions from FGFR1-3 and suggesting a less prominent role for α-Klotho-dependent FGFR signaling in the CNS. Conversely, inhibition of FGFR4 or calcineurin signaling in the brain restored excess Pi-mediated sympathoexcitation and hypertensive responsiveness, thereby identifying a noncanonical FGFR4-calcineurin pathway as a key mediator of central FGF23 effects (Figure 2). This restoration coincided with a selective increase in brainstem levels of a calcineurin-activity-dependent regulator of calcineurin 1 isoform 4, suggesting enhanced calcineurin activity in the brainstem during excess Pi intake [53,54].

Consistent with the above findings, inhibition of the canonical pathway in the brain had no significant impact on the abnormal response to passive muscle stretch or IA capsaicin administration during high Pi loading, whereas central noncanonical pathway inhibition exerted significant inhibitory effects. Moreover, intravenous blockade of pan-FGFR(1–4) signaling failed to attenuate the high-Pi-diet-induced neurogenic hypertensive responses, indicating that peripheral FGFR signaling does not contribute to the development of a neurogenic hypertensive phenotype caused by excess Pi loading. These findings establish a role for the noncanonical FGFR4-mediated pathway in the brain in mediating high-Pi-induced sympathetic overactivity and hypertensive responses. The evidence supporting this pathway is summarized in Table 1. Nonetheless, these insights are largely based on animal studies, and direct evidence defining the role of brain FGFR signaling in humans is yet to be investigated. Further experimental studies are required to determine which brain regions (e.g., NTS and RVLM) are involved in these effects.

Existing experimental findings support that FGF23-induced hypertension and LVH are mediated by distinct mechanisms. Previous studies have proposed the role of FGF23 in promoting LVH via FGFR4-calcineurin signaling in the cardiomyocytes [48,49,50,51]. In mice with FGFR4 deletion in the cardiomyocytes, FGF23-induced LVH was attenuated, whereas FGF23-induced hypertension persisted at the levels comparable to FGF23-treated wild-type mice [51]. Therefore, high dietary Pi intake may contribute to adverse cardiovascular remodeling via both BP-dependent and BP-independent mechanisms.

### 3.3. Role of Klotho in Sympathetic Nervous System

Apart from brain FGF23-FGFR4 signaling, Pi may stimulate sympathetic activation by altering Klotho expression and soluble Klotho levels. A high-Pi-diet downregulates Klotho expression in the kidneys and reduces soluble Klotho levels in mouse serum [55]. Increasing evidence has linked Klotho deficiency to the pathogenesis of hypertension [56]. Polymorphism in the *KLOTHO* gene is associated with human hypertension [57,58].

Previous studies have suggested potential involvement of Klotho within the CNS in BP regulation. Klotho is also produced in the choroid plexus of the brain [59,60], and soluble Klotho has been detected in the CSF of humans [61]. Klotho suppresses neuronal activity of presympathetic neurons in the rat RVLM in vitro [42]. Silencing Klotho expression in the brain choroid plexus generates stress (cold exposure)-induced hypertension in rats [62]. This is accompanied by increased plasma levels of norepinephrine, suggesting heightened sympathetic activation.

Nonetheless, previous studies have not addressed the role of Klotho in regulating SNA during dietary Pi loading or during FGF23 administration directly. As mentioned earlier, since disruption of Klotho interaction with FGF23 via ICV C-terminal peptide administration fails to suppress sympathetic activity and BP responses [33], it is unlikely that Klotho plays a major role in the central neural control of BP during excess Pi intake. Further studies are needed to elucidate the role of Klotho in sympathetic regulation during high Pi loading.

### 3.4. Renal Mechanisms

Excess Pi (extracellular or intracellular) exerts toxic effects in the kidneys [63]. High Pi exposure has been shown to induce mitochondrial injury, increase mitochondrial reactive oxygen species (ROS) production, and decrease cellular ATP levels, thereby contributing to cell death in renal proximal tubular cells [64]. A high Pi milieu directly induces cellular senescence, injury, and epithelial–mesenchymal transition, as well as enhanced H_2_O_2_-induced senescence and increased oxidative stress in the kidney tubule epithelial cell line in vitro. This direct phosphotoxicity is abrogated by Klotho administration [65]. High Pi concentration has also been shown to induce cell proliferation and lower cell viability, leading to cell death in a human embryonic kidney cell line in a dose-dependent manner [66]. Consistent with these findings, excess Pi itself contributes to organ injury and dysfunction, even in the absence of CKD [67,68].

Whether this Pi-induced organ impairment is mediated by FGF23-FGFR signaling pathways remains to be defined. In mice with kidney-specific deletion of FGFR1, FGF23 administration did not alter serum Pi levels. However, in global FGFR4 knockout mice, FGF23 treatment significantly reduced serum Pi, presumably acting through the intact FGFR1 [45]. Double mutant mice with the kidney-specific FGFR1 deletion and global FGFR4 knockout exhibited elevated serum Pi and FGF23 levels compared to wild-type and FGFR4 knockout mice [46]. These findings indicate that FGFR1 plays a predominant role in mediating FGF23-induced renal Pi regulation, while contribution of FGFR4 is less significant. FGFR4 ablation in CKD mice did not significantly affect kidney injury, survival, serum Pi and FGF23 levels, or renal fibrosis compared to wild-type CKD mice, indicating that FGFR4 may not promote CKD progression [69].

Klotho deficiency induced by high Pi loading may also alter renal Na^+^ regulation via action of aldosterone, which promotes renal distal Na^+^ retention and affects BP. Haplodeficient Klotho^+/−^ mice showed concomitant increases in circulating aldosterone levels and systolic BP, which coincided with upregulation of the Na^+^:Cl^−^ co-transporter (NCC) in kidneys [70]. These adverse effects of klotho deficiency were abolished by mineralocorticoid receptor antagonist eplerenone treatment, suggesting aldosterone mediated volume expansion and hypertension. In contrast, Klotho-hypomorphic mice (*Kl/Kl*) exhibited extracellular volume depletion and reduced systolic BP with secondary increases of antidiuretic hormone and aldosterone release [71]. This indicates that lack of Klotho leads to hyperaldosteronism without necessarily causing hypertension. These contrasting findings highlight the need for further research to clarify the interplay between Klotho, aldosterone, and BP regulation.

Increased renal renin production mediated by low 1,25(OH)_2_D [72,73], the active form of vitamin D, is another potential mechanism underlying FGF23-related hypertension during high Pi loading. Pi directly, and also indirectly via FGF23, suppresses 1-α hydroxylase activity, the key enzyme in vitamin D activation, thereby reducing 1,25(OH)_2_D production [74,75]. Whether FGF23 excess and Klotho deficiency stimulates the renin–angiotensin–aldosterone system to promote Pi-induced hypertension remains unproven.

### 3.5. Endothelial Dysfunction and Vascular Remodeling

A high Pi milieu acutely impairs endothelium-dependent dilation, in rodent aortic rings [76] and mesenteric arteries [77]. Similarly, a high Pi diet for 16 days impaired endothelium-dependent dilation in rat aortas [78], and longer Pi exposure (12 weeks) exerted a similar impact in mouse resistance arteries [79]. These adverse effects are thought to result from increased ROS production and decreased endothelial nitric oxide synthase (eNOS) activation, leading to reduced vasodilator nitric oxide (NO) bioavailability [77,78]. Notably, the inorganic Pi transporter PiT-1 mediates the high-Pi-induced reduction in eNOS activity via mitochondrial oxidative stress in endothelial cells [79].

Human studies investigating the effects of high Pi loading on endothelial function have focused on brachial artery flow-mediated dilation (FMD), as a surrogate measure, supporting the model of Pi impairing endothelial function. In healthy volunteers, excess Pi intake acutely attenuated FMD [80,81], and a 2-week intervention induced similar effects [77].

Elevated circulating FGF23 levels have been associated with endothelial dysfunction in the general population [82,83]. However, experiments investigating direct effects of FGF23 have yielded inconsistent results. Endothelial cells express FGFR1/2/3, but not FGFR4, with FGFR1 being the most abundant [84]. Exogenous FGF23 administration for 7 days impaired endothelium-dependent dilation in mouse mesenteric artery [85]; however, short-term exposure of mouse gracilis [85] or mesenteric arteries to FGF23 [86] had no significant effect. Short-term exposure to FGF23 directly induces endothelial dysfunction in mouse aortas by reducing NO bioavailability [87] in a Klotho-independent manner. In the presence of anti-Klotho neutralizing antibody, however, FGF23 promoted ROS formation and oxidative stress, resulting in reduced NO bioavailability in human coronary artery endothelial cells [88]. These inconsistent findings suggest that a high Pi environment, rather than FGF23 excess per se may exert a greater impact on endothelial dysfunction.

The expression of Klotho in the vasculature remains controversial [86,87,89,90,91]. Klotho-deficient mice, characterized by decreased soluble Klotho exhibited endothelial dysfunction in aortic and resistance arteries, which was restored by parabiosis with wild-type mice [92,93], indicating the important role of soluble Klotho in maintaining endothelial function. Future studies are needed to clarify the role of FGF23 excess and Klotho deficiency on endothelial function and BP regulation during dietary Pi loading.

In addition to impairment of endothelial function, long-term dysregulation of Pi and FGF23 may also contribute to vascular remodeling. Arterial stiffening resulting from vascular calcification is another potential mechanism linking Pi and FGF23 to hypertension. High Pi milieu can directly induce “osteogenic” transformation (a term used to indicate expression of osteogenic genes but not true osteogenesis) of vascular smooth muscle cells (VSMCs) in vitro, an early step for vascular calcification and stiffening [94,95,96,97]. High Pi upregulates Runx2, a key osteogenic transcription factor, inducing human VSMC calcification via PiT-1 [96]. Pi also increases rat aortic VSMC oxidative stress [98,99], a potent stimulus for Runx2 upregulation [100] and subsequent VSMC calcification [101,102].

Human studies assessing Pi intake effects on arterial stiffness have used aortic pulse wave velocity (PWV) as an indirect measure. Excess Pi consumption in healthy volunteers for 2 or 6 weeks did not significantly affect aortic PWV [15,81]. However, interpretation of the data is limited by small sample size, short intervention durations, and the large coefficients of variation inherent to noninvasive arterial stiffness measures.

Studies examining the role of FGF23 in vascular calcification have yielded conflicting results. In VSMCs, FGFR isoform expression is similar to that found in endothelial cells [84]. Treatment of rat aortic rings and VSMCs with FGF23 for 6 days enhanced Pi-induced vascular calcification [103]. These effects were not prevented by the presence of Klotho. In contrast, studies in human VSMCs reported that FGF23 neither augmented Pi-induced calcification nor had any effect, regardless of soluble Klotho [90]. Moreover, FGF23 alone did not induce vascular calcification in mouse aortas [90] or rat, human [90,103], or bovine VSMCs after 10 days of incubation [86].

Notably, phenotypic switching of VSMCs from contractile to synthetic has been linked to hypertension [104]. The synthetic VSMC phenotype is characterized by decreased contractile protein expression, increased extracellular proteins, and enhanced proliferation and migration abilities. FGF23 induced this phenotype transition, presumably via FGFR1 signaling, decreasing expression of α-smooth muscle actin (α-SMA), elastin, and myosin heavy chain (markers of the contractile phenotype), while increasing matrix metallopeptidase 9 (MMP9, an extracellular matrix proteinase) and proliferation in human aortic VSMCs [105]. Exogenous FGF23 administration increased vascular wall thickness, accompanied by VSMCs exhibiting the synthetic phenotype in rats [104]. Furthermore, aortic rings from rats treated with excess FGF23 exhibited increased arterial stiffness [104]. These findings suggest that apart from vascular calcification, FGF23 may contribute to vascular dysfunction by promoting arterial stiffening. Whether FG23-mediated VSMC phenotype changes and arterial stiffness causally contribute to BP regulation remains to be determined.

## 4. Phosphotoxicity and Hypertension in CKD

Sympathetic overactivation [106] and hypertension [107] are common in individuals with CKD, and they exhibit exaggerated exercise pressor responses [108,109]. In an animal model of CKD, sympathetic overactivity is a significant contributor to the abnormal hypertensive responses during simulated exercise [110].

Persistent Pi retention and excess circulating FGF23 are common in CKD [111]. As kidney function declines, renal Pi excretion becomes progressively impaired, contributing to the initiation of Pi retention. Increased FGF23 secretion into the circulation is necessary to counteract this retention and maintain normal Pi balance. However, with CKD progression, renal Klotho deficiency reduces FGFR1/α-Klotho signaling and leads to renal FGF23 resistance. This impaired responsiveness contributes to further increases in circulating FGF23 levels, generating a maladaptive cycle in which both Pi and FGF23 remain chronically elevated [111], and therefore, each is a potential therapeutic target for hypertension in CKD.

Nevertheless, direct evidence linking these factors to sympathetic and BP dysregulation in CKD are limited. In hemodialysis patients, serum Pi is independently associated with pre-hemodialysis BP and total peripheral resistance [112]. However, the long-term impact of dietary Pi restriction on BP in hemodialysis is unclear. This uncertainty is partly due to the difficulty of sustaining dietary Pi restriction in the presence of hidden Pi additives in processed foods and the modest effects of phosphorus binders in lowering serum Pi. To date, there are no clinical data evaluating the use of FGF23-neutralizing antibodies in patients with CKD. This lack of clinical evidence is likely attributable to findings from advanced CKD animal models in which complete neutralization of FGF23 using a monoclonal antibody led to high mortality [113], possibly due to Pi retention and other factors. In contrast, studies in CKD animals have shown that peripheral blockade of FGF23 signaling, such as FGFR4 [49] or calcineurin [114], attenuates adverse cardiac remodeling, but does not reduce high BP. These findings indicate that the effects of peripheral inhibition of FGF23 signaling are BP-independent; therefore, targeting peripheral FGF23 pathways may not adequately address hypertension in CKD.

Notably, recent evidence indicates that FGF23 can enter the CNS [33], and this translocation is also potentially facilitated in CKD as hemodialysis patients have been shown to exhibit increased blood–brain permeability [115]. This notion is further supported by in vitro studies demonstrating reduced expression of the tight junction protein claudin-5 and the adherens junction protein platelet endothelial cell adhesion molecule-1, both of which are involved in maintaining blood–-brain barrier integrity, in the brain endothelial cells of CKD rodents [116]. Considering that our recent studies demonstrated that central, rather than peripheral pathogenic intermediates, FGFR activation mediates high-Pi-diet-induced sympathetic overactivation and hypertensive responses, future research needs to focus on clarifying these central pathways.

## 5. Conclusions and Future Directions

Hypertension remains a major global health challenge with dietary Pi and FGF23 emerging as important contributors to elevated BP. High Pi intake directly and indirectly, through its hormonal response of elevated FGF23, promotes vascular abnormalities, stimulates sympathetic nervous system activation, and possibly enhances renal Na+ retention. Recent evidence in rats indicates that peripheral FGF23 crosses the blood–brain barrier and stimulates brain FGFR4-calcineurin signaling, leading to sympathetic overactivation and hypertensive responses mediated by excess Pi intake.

While key components of this pathway are supported by direct experimental evidence in rodent models, species differences in Pi metabolism, together with the limited availability of direct evidence for FGF23-FGFR signaling within the human CNS, warrant cautious interpretation when translating these findings to humans.

Nevertheless, these findings suggest that targeting brain FGF23 signaling, either by lowering circulating FGF23 levels or by inhibiting FGFR4 activation, may offer novel approaches to mitigate sympathetic overactivation and hypertension linked to excess Pi intake. In addition to FGF23 signaling, Pi may stimulate sympathetic activation through mechanisms related to reduced Klotho levels; however, the role of CNS soluble Klotho outside its role as an FGF23 co-receptor in sympathetic regulation during high Pi loading remains unexplored and warrants further investigation.

These mechanisms are potentially relevant in CKD, where persistent Pi retention, elevated FGF23, suppressed Klotho, sympathetic overactivation, and hypertension are commonly observed. Therefore, future studies examining whether antagonism of brain FGF23-FGFR4 signaling and restoration or modulation of Klotho can alleviate sympathetic dysregulation in CKD may provide important mechanistic insights and, most importantly, opportunities for intervention. Overall, this review summarizes and enhances our understanding of the cardiovascular effects of brain FGF23-FGFR4 signaling and provides insights into its potential role as a therapeutic target for developing treatment strategies for hypertension associated with high Pi intake and CKD.

## Figures and Tables

**Table 1:** Summary of evidence linking a high Pi diet, central FGF23 signaling, sympathetic overactivation, and hypertension.

Exposure/Condition	Outcome	Study Model	Evidence Type
Pi additives intake	↑ BP (positive association)	Human	Indirect
High Pi intake	↑ BP	Human	Direct
High Pi diet	↑ BP	Mouse/Rat	Direct
High Pi diet	↑ SNA and ↑ BP responses	Rat	Direct
Circulating FGF23 levels	↑ BP (positive association)	Human	Indirect
High Pi diet	↑ circulating and brain FGF23 levels	Rat	Direct
IV labeled FGF23	Detected in the choroid plexus and brainstem regions	Rat	Direct
ICV FGF23 injection	↑ SNA and ↑ BP	Rat	Direct
ICV FGFR4 inhibitor *	↓ SNA and ↓ BP responses	Rat	Direct
ICV calcineurin inhibitor *	↓ SNA and ↓ BP responses	Rat	Direct

BP, blood pressure; FGF23, fibroblast growth factor 23; FGFR, fibroblast growth factor receptor; ICV, intracerebroventricular; IV, intravenous; Pi, phosphate; SNA, sympathetic nerve activity. An upward arrow indicates an increase, whereas a downward arrow indicates a decrease. * Pharmacological interventions were performed in animal models subjected to a high-Pi diet.

## Data Availability

No new data were created or analyzed in this study. Data sharing is not applicable to this article.

## Abbreviations

The following abbreviations are used in this manuscript:

ABP	Arterial blood pressure
BP	Blood pressure
CKD	Chronic kidney disease
CNS	Central nervous system
CSF	Cerebrospinal fluid
eNOS	Endothelial nitric oxide synthase
EPR	Exercise pressor reflex
FGF23	Fibroblast growth factor 23
FGFRs	Fibroblast growth factor receptors
FMD	Flow-mediated dilation
IA	Intraarterial
ICV	Intracerebroventricular
LVH	Left ventricular hypertrophy
NO	Nitric oxide
NTS	Nucleus tractus solitarius
Pi	Phosphate
PWV	Pulse wave velocity
ROS	Reactive oxygen species
RVLM	Rostral ventrolateral medulla
SNA	Sympathetic nerve activity
VSMCs	Vascular smooth muscle cells
